# Elimination of Lead by Iodide of Potassium

**Published:** 1854-01

**Authors:** J. Outram

**Affiliations:** Lecturer on Chemistry in the New York Preparatory School of Medicine


From the Medical Times.

*Elimination of Lead by Iodide of Potassium.* Reported to the Biological Society by J. Outram, Jun, Lecturer on Chemistry in the New York Preparatory School of Medicine.

The value of iodide of potassium, as a therapeutical agent in cases of lead and mercurial poisoning, is now well known, many cases having been cured by this treatment alone, in our hospitals. M. Melsens (the originator of this treatment) has given several cases of both lead and mercurial poisoning, which he had treated successfully by this means. He was of opinion that the metal was acted upon by the iodide of potassium converted into a soluble salt, and eliminated through the kidneys. To prove this to be the case, the metal must of course be found in the urine. This M. Melsens did not show, as he did not examine the urine chemically.

I have lately had an opportunity of examine the urine of several patients at the City Hospital, who have been under the above treatment for lead disease, and the experiments have entirely corroborated M. Melsens' theory, viz. :

That the lead *is not* eliminated before treatment ; and,

That it is eliminated in the urine after treatment.

The following is the process to which I subjected the urine. I evaporated it to dryness, and burned the residue until all the organic matter was driven off. This residue I boiled in dilute nitric acid, filtered, and then precipitated the lead by a stream of sulphureted hydrogen gas. Where the metal was present, it was thrown down as a black sulphuret.

I examined a number of samples of urine before the patients had been put on M. Melsens' treatment, and could not detect any trace of metal; while in those examined after treatment, the evidences of the metal were well marked. Sometimes, however, the lead could not be detected until the patient had been under treatment for some time. In most of the cases in which I detected the metal, the patients had been under treatment for at least four days; but in one case, which I examined every second or third day, from the time of his admission into the Hospital, it was about two weeks from the time of his being put on treatment till the time I first detected the metal in his urine. The dose of iodide of potassium that was given in those cases was ʒi daily, in divided doses.

There was one patient to whom iodide of potassium was given for another case. After a few days, the characteristic blue line appeared on the gums, and, in a day or two afterwards, I detected lead in his urine.

The quantity of urine examined each time was not less than six ounces. I have examined twelve cases from the Hospital, and three or four from private practice, and all of them with highly satisfactory results.

